# Defining Integrated Knowledge Translation and Moving Forward: A Response to Recent Commentaries

**DOI:** 10.15171/ijhpm.2017.15

**Published:** 2017-02-08

**Authors:** Anita Kothari, Chris McCutcheon, Ian D. Graham, for the IKT Research Network

**Affiliations:** ^1^School of Health Studies, University of Western Ontario, London, ON, Canada.; ^2^Integrated Knowledge Translation Research Network, Ottawa Hospital Research Institute, Ottawa, ON, Canada.; ^3^School of Epidemiology, Public Health and Preventive Medicine, University of Ottawa, Ottawa, ON, Canada.

Integrated knowledge translation (IKT) is a model of collaborative research, where researchers work with knowledge users who identify a problem and have the authority to implement the research recommendations. Knowledge users have unique expertise pertaining to the research topic, including knowledge of the context and the potential for implementation. Researchers bring methodological and content expertise to the collaboration. Implicit in this approach is the sharing of power between researchers and knowledge users. Sometimes referred to as the co-production of knowledge, this new way of working suggests that the synergies derived from the collaboration will result in better science; more relevant and actionable research findings; increased use of the findings in policy or practice; and mutual learning. An evaluation of knowledge translation funding programs at the Canadian Institutes of Health Research demonstrated that researchers and knowledge users co-producing research were more likely to report improving the health of Canadians, creating more effective health services or products and strengthening the Canadian healthcare system than researchers who do not work with knowledge users.^1^

Interest in IKT as a strategy for accelerating the uptake and impact of research is growing as demonstrated by the recent publication of articles and commentaries in this and other journals.^2-5^ Rycroft-Malone et al^6^ note that, while promising, the IKT approach is not without its challenges. The authors highlight issues of power, politics, and perceptions that require careful attention if an IKT approach is to be successful. Collaborations, they argue, require prompting and support. The authors also suggest that certain personable qualities, such as being tolerant of less structure whilst maintaining methodological standards, are required of individuals participating in IKT partnerships. A related commentary was offered by Cooke et al^7^ in which they address the issue of power. In particular, these authors argue that the co-production process ought to be visible, and that actionable outputs (in the form user-oriented products, like toolkits) representing this joint effort are necessary. The authors present their experience with the use of design as one way to flatten hierarchy and show co-produced knowledge artefacts.

A recent scoping review^8^ about IKT related to organizational and system-level decision-making identified some notable knowledge gaps in the literature. A detailed understanding of IKT strategies and models is needed so that they can be linked to outcomes. The review also demonstrated minimal theoretical development in the area. Finally, the review showed that we do not yet understand how decision-makers ought to be engaged to achieve optimal outcomes. Similar knowledge gaps were found in a review by Camden et al.^9^ As IKT scholars consider and prioritize research questions, it might be useful to remember that different stakeholders will assign the gaps varying importance; what a funder needs to know about IKT (eg, “how can funders incentivize researchers to engage in IKT research?”) will be different than what a researcher will want to know (eg, “what theory can be used to understand IKT processes?”). Both IKT researchers and knowledge users are interested in how to define and measure IKT outcomes.

In 2016, we launched a 7-year program of research funded by a Canadian Institutes of Health Research Foundation Grant called the Integrated Knowledge Translation Research Network. The program, housed at the University of Ottawa, began as a collaboration of over 40 researcher and knowledge-user co-investigators, but it has evolved into a network so that we can build additional linkages and collaborations with the many people within and outside Canada committed to better understanding and using the IKT approach to research.

The first order of business for the network is to achieve clarity on the differences between IKT and other collaborative research approaches and to delineate the benefits of that clarity. To that end we have begun a conceptual analysis of multiple collaborative research traditions. This is a starting point, but much more needs to be done to develop the theoretical gaps in IKT that we have already alluded to. We want to learn how successful IKT research projects operate and what the mechanisms are, which is why we began a realist review on the IKT research process late last year. Because IKT involves multiple institutions and stakeholders, we will be collaborating with funders and health-system organizations to determine what conditions will foster the best collaborations and impacts. For example, we have launched projects exploring how organizations decide when and how to partner with researchers. Of course, we want to be able to answer the question, “Does it work?” In the coming years we will launch projects aimed at measuring the impacts of IKT research. We will not be able to fill all of the knowledge gaps, but we have six years to train a new generation of IKT researchers who will answer the new and outstanding questions.

## Acknowledgments

IDG is a recipient of an inaugural Canadian Institutes of Health Research Foundation Grant [FDN #143237]. Current members of the IKTR Network are: Gonzalo Alvarez, Beth Beaupre, Ingrid Botting, Jamie Brehaut, Krista Connell, Sandra Dunn, Jeanette Edwards, Shannon Fenton, Ann Gagliardi, Ian D. Graham, Jeremy Grimshaw, Wendy Gifford, Bev Holmes, Michael Hillmer, Russell Ives, Ian Jones, Monika Kastner, Anita Kothari, Sara Kreindler, John Lavis, Karen Lee, France Legare, Debra Lynkowski, Martha MacLeod, Theresa Montini, Jo Rycroft-Malone, Patrick Odnokon, Sheldon Permack, Jonathan Salsberg, Yves Savoie, Gayle Scarrow, Robert Sheldon, Ann Sprague, Janet Squires, Dawn Stacey, Sharon Straus, Anthony Tang, Cathy Ulrich, Pam Valentine, Christina Weise, George Wells, Brock Wright.

## Ethical issues

Not applicable.

## Competing interests

Authors declare that they have no competing interests.

## Authors’ contributions

AK drafted the response. All authors then edited, contributed written text and reviewed the final version of the article. IDG was an author of the article that is the subject of the commentaries.

## Authors’ affiliations

^1^School of Health Studies, University of Western Ontario, London, ON, Canada. ^2^Integrated Knowledge Translation Research Network, Ottawa Hospital Research Institute, Ottawa, ON, Canada. ^3^School of Epidemiology, Public Health and Preventive Medicine, University of Ottawa, Ottawa, ON, Canada.
